# Supplementary material for: Atomic structures of anthrax toxin protective antigen channels bound to partially unfolded lethal and edema factors
Source: Nat Commun. 2020 Feb 11;11:840. doi: 10.1038/s41467-020-14658-6 (PMC7012834; doi:10.1038/s41467-020-14658-6)
Supplement: Supplementary file 1 — Supplementary Information [file 41467_2020_14658_MOESM1_ESM.pdf]

Supplementary Information

Atomic structures of anthrax toxin protective antigen channels bound to partially unfolded lethal and edema factors

Nathan J. Hardenbrook, Shiheng Liu, Kang Zhou, Koyel Ghosal, Z. Hong Zhou, Bryan A. Krantz

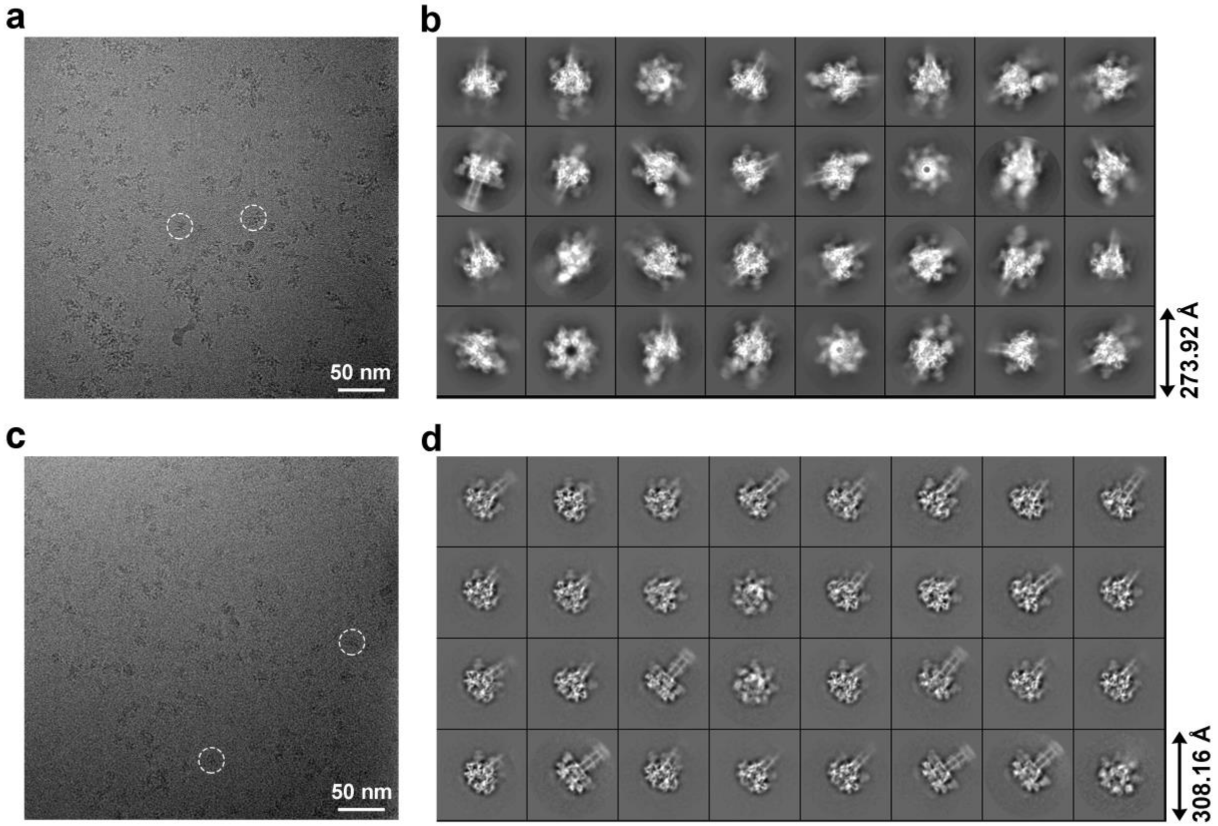

Supplementary Figure 1. Cryo-EM image and 2D classification of LF-bound and EF-bound PA channels.

a, A cryo-EM micrograph of the EF-bound PA channels after drift correction. **b,** Representative 2D class averages of EF-bound PA channels obtained in RELION. **c,** A cryo-EM micrograph of the LF-bound PA channel after drift correction. **d,** Representative 2D class averages of LF-bound PA channel obtained in RELION. The scale bars in panels a and c are 50 nm.

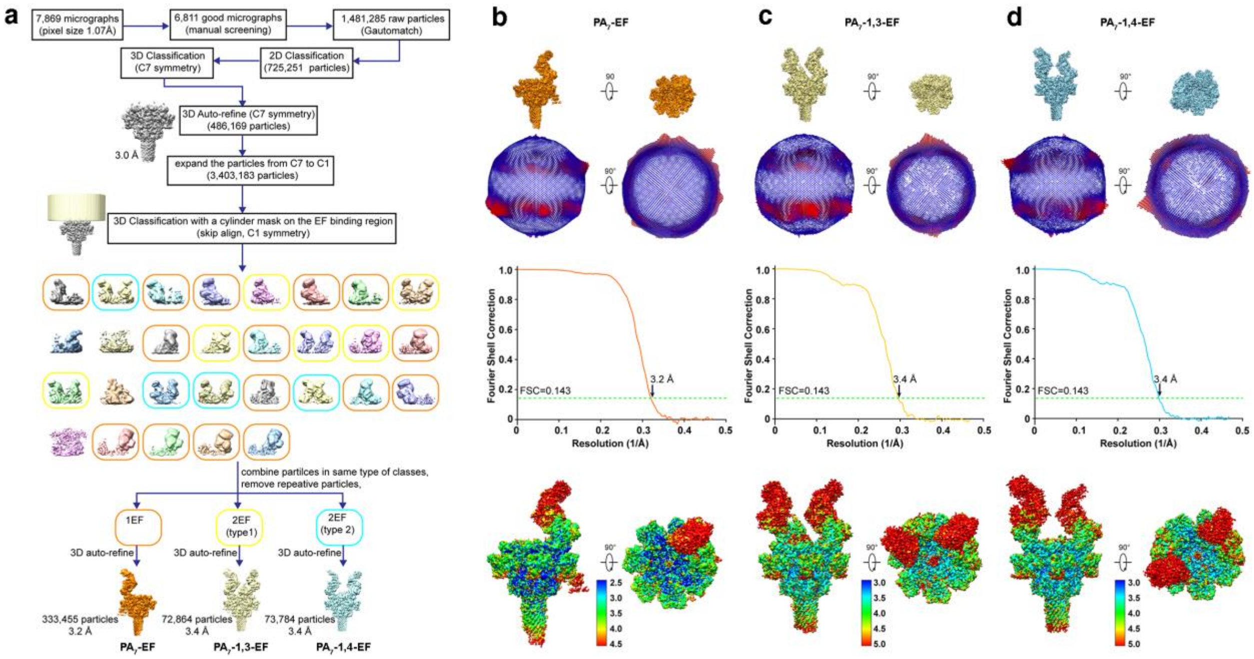

Supplementary Figure 2. Structural determination process for EF-bound PA channels.

a, Data processing workflow. See Materials and Methods for more details. **b-d,** Upper panels: angular distribution for all particles used for the final maps; middle panels: FSC as a function of spatial frequency demonstrating the resolution for the final reconstruction; lower panels: *Resmap* local resolution estimation.

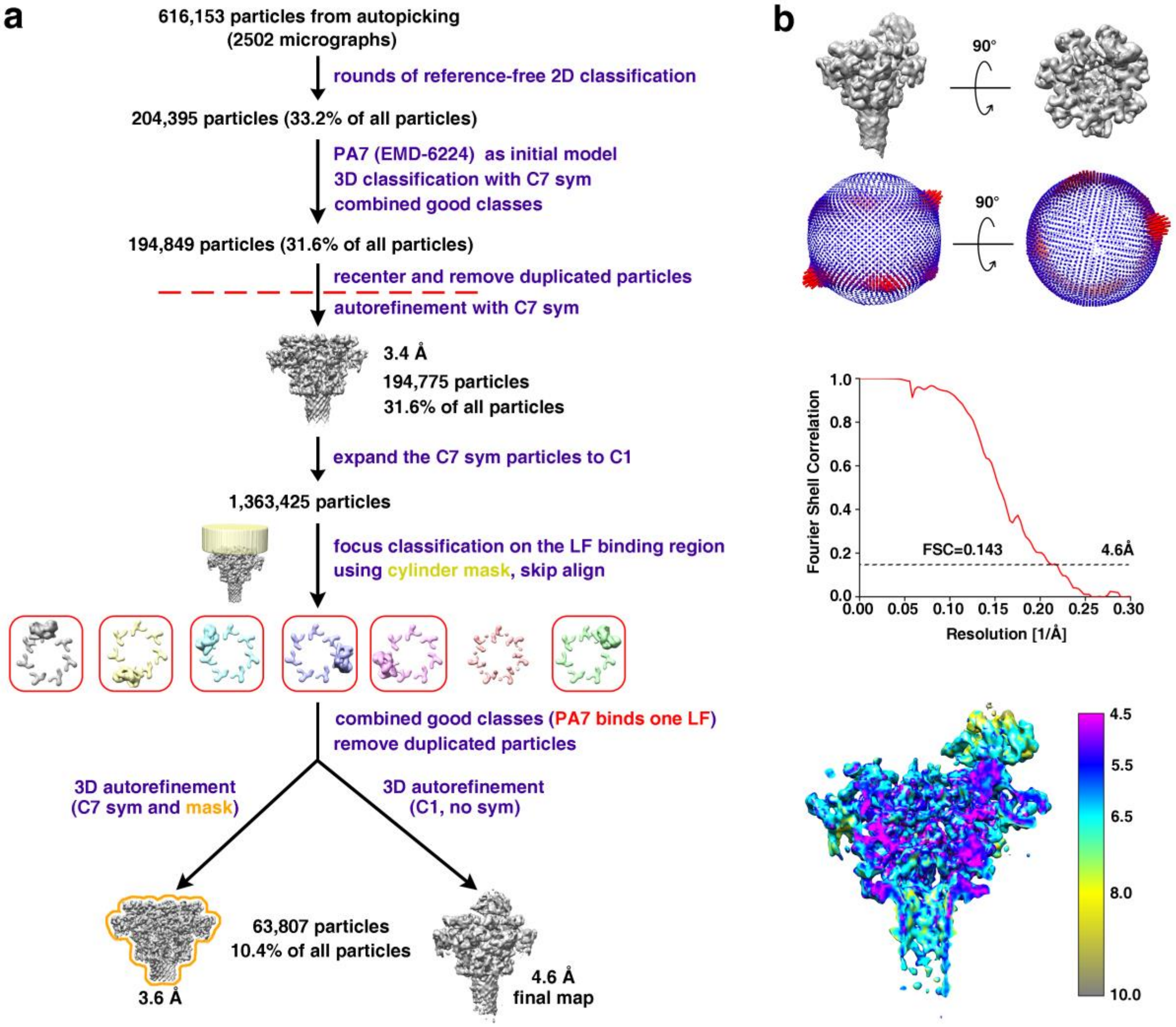

Supplementary Figure 3. Structural determination process for the LF-bound PA channel. **a,** Data processing workflow. For processing above the red dash line, the particle images were binned to a pixel size of 2.14 Å. The rest of processing was performed with a pixel size of 1.07 Å. See Materials and Methods for more details. **b,** Upper panel: angular distribution for all particles used for the final map; middle panel: FSC as a function of spatial frequency demonstrating the resolution for the final reconstruction; lower panel: *Resmap* local resolution estimation.

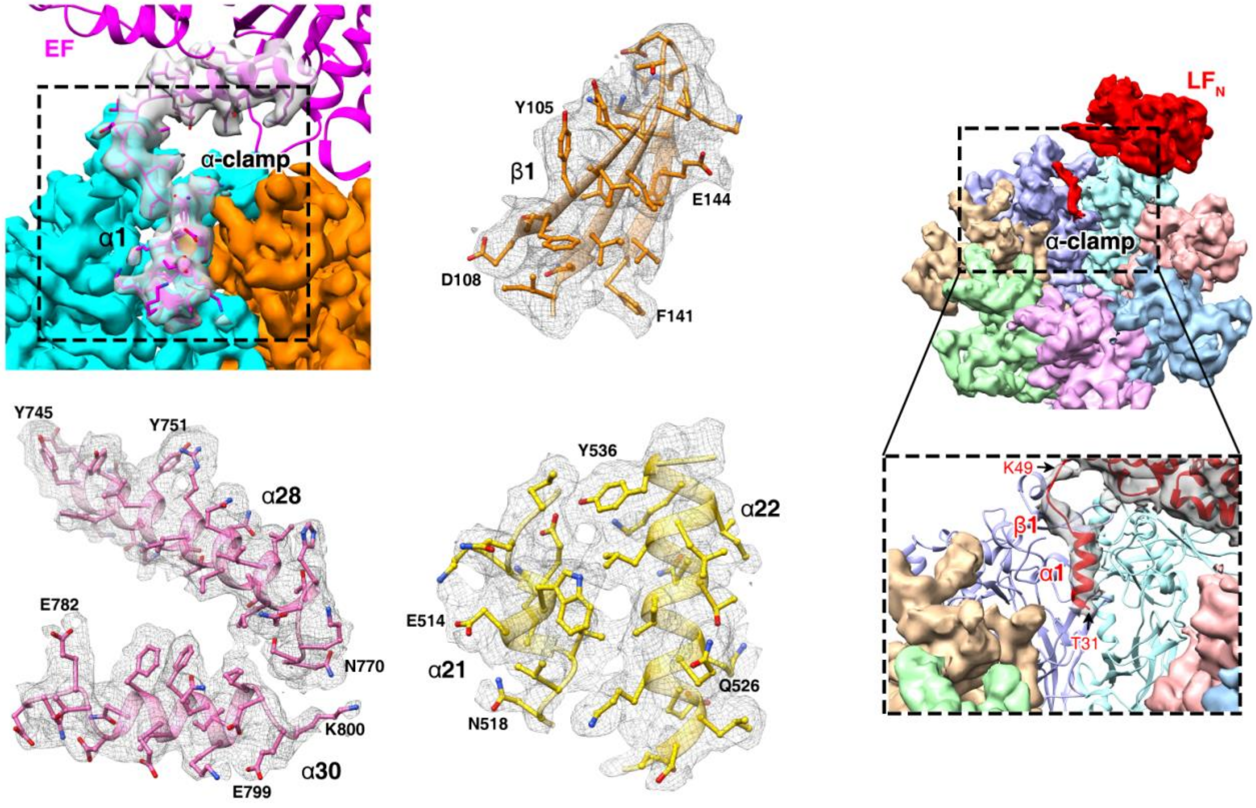

EF in PA$_7$-EF **LF in PA$_7$-LF**

Supplementary Figure 4. Representative cryo-EM density maps for both EF and LF complexed with PA channels.

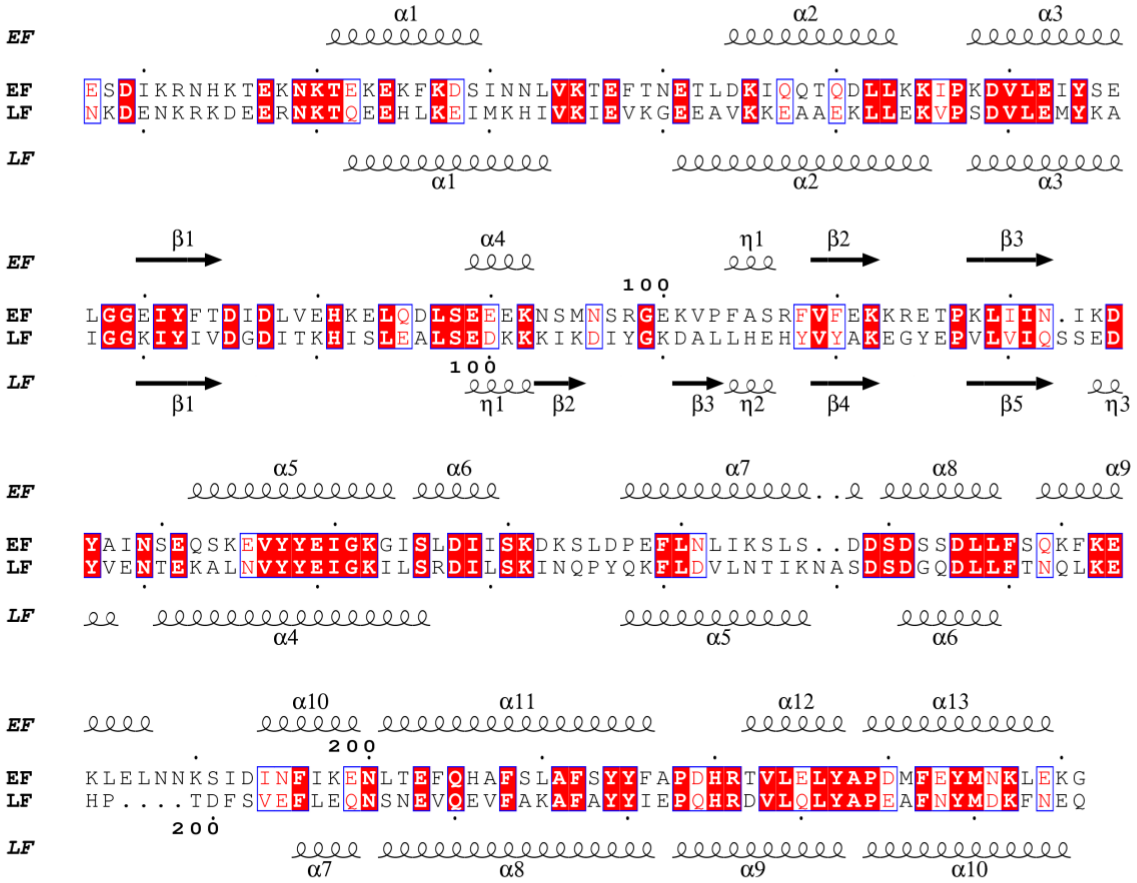

Supplementary Figure 5. Sequence conservation analysis between LF and EF.

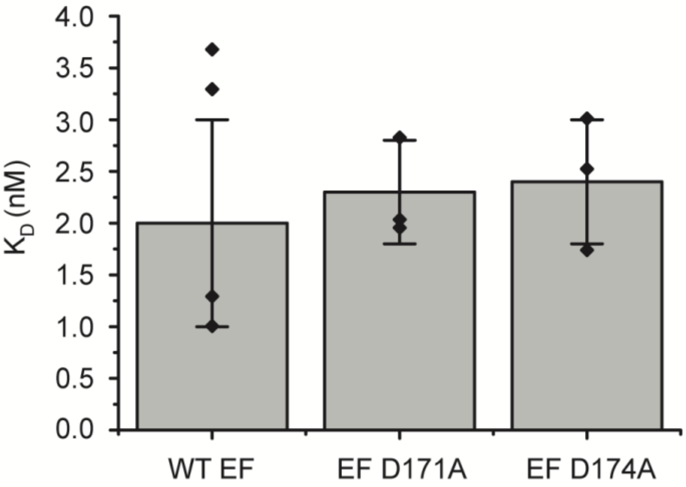

Supplementary Figure 6. Binding affinities of EF binding interface mutants.

Ensemble lipid bilayer measurements of binding affinities. Binding of EF and salt bridge mutants to PA channels at symmetric pH 7.0 and asymmetrical KCl (100 mM [added KCl]$_{cis}$, 0 mM [added KCl]$_{trans}$). At the stabilization of the ensemble current at about 4000-5000 pA, EF or salt bridge mutants were added in small increments to the cis and allowed to equilibrate. Final current values were determined. Curves were fit in ORIGIN9. Error bars represent standard deviation of the equilibrium dissociation constant (K_D) for three or four independent measurements.

Supplementary Table 1. EF Mutagenesis Primers

Primer Name	Sequence
EF D171A Forward	5'-GACTAAATAAAAGGT-3'
EF D171A Reverse	5'-TAAGAGTTTAAGCGA-3'
EF D174A Forward	5'-CTTTAAATTTTTGAC-3'
EF D174A Reverse	5'-TTAAGCGATGATAGT-3'

Supplementary Table 2. CryoEM data collection and refinement statistics.

**Data collection**	LF complex	EF complex		
	PA_7-LF	PA_7-EF	PA7-1,3-EF	PA7-1,4-EF
EM equipment	FEI Titan Krios	FEI Titan Krios		
Voltage (KV)	300	300		
Detector	Gatan K2	Gatan K2		
Pixel size (Å)	1.07	1.07		
Electron dose (e^-/Å^2)	62.9	60.2		
Defocus range (µm)	-1.5 ~ -3.0	1.5 ~ -3.0		
**Reconstruction**				
Software	RELION2.1	RELION2.1		
Number of used particles	63,807	333,455	72,864	73,784
Accuracy of rotation (°)	2.51	1.62	1.36	1.41
Accuracy of translation (pixels)	1.70	0.76	0.71	0.72
Map sharpening B-factors (Å^2)	-169.7	-80	-80	-80
Resolution FSC 0.143 (Å)	4.6	3.2	3.4	3.4
**Model building**				
Software	COOT	COOT	COOT	COOT
**Refinement**				
Software	PHENIX	PHENIX	PHENIX	PHENIX
Resolution (Å)	4.6	3.2	3.4	3.4
R-factor	0.37			
Number of protein residues	4,154	3,681	4,401	4,401
Map CC	0.81	0.86	0.84	0.85
R.m.s deviations				
Bonds length (Å)	0.01	0.01	0.01	0.01
Bonds angle (°)	1.15	1.17	0.90	0.94
Ramachandran plot statistics (%)				
Preferred	96.67	95.49	93.30	92.72
Allowed	3.33	4.51	6.69	7.18
Outlier	0	0	0	0.1
Rotamers outliers (%)	0.40	0.29	0.21	0.35
C-beta deviations	0	0	0	0
Clash score	6.78	4.83	5.34	5.24
MolProbity score	1.59	1.57	1.72	1.74
PDB code	6PSN	6UZB	6UZD	6UZE
EMDB code	EMD-20459	EMD-20955	EMD-20957	EMD-20958
