# Supplementary material for: Atomic structures of anthrax toxin protective antigen channels bound to partially unfolded lethal and edema factors
Source: Nat Commun. 2020 Feb 11;11:840. doi: 10.1038/s41467-020-14658-6 (PMC7012834; doi:10.1038/s41467-020-14658-6)
Supplement: Supplementary file 3 — Description of Additional Supplementary Files [file 41467_2020_14658_MOESM3_ESM.pdf]

Description of Additional Supplementary Files

File Name: Supplementary Movie 1

Description: CaM-bound and PA_7-bound EF forms morph. Morph movie, calculated using the “morph conformations” function in UCSF Chimera, of the closed and open states. This morph is not representative of the exact motion of the mechanism, but is intended to give a sense of scale for the dramatic conformational shift.
